# Immature platelet fraction predicts early marrow recovery after severe chemotherapy associated neutropenia

**DOI:** 10.1038/s41598-023-30469-3

**Published:** 2023-02-27

**Authors:** Christina Salvador, Andreas Meryk, Benjamin Hetzer, Caroline Bargehr, Gabriele Kropshofer, Bernhard Meister, Markus Anliker, Roman Crazzolara

**Affiliations:** 1 Division of Hematology and Oncology, Department of Pediatrics I, Medical University of Innsbruck, Innsbruck, Austria; 2 Central Institute for Medical and Chemical Laboratory Diagnosis, Medical University of Innsbruck, Innsbruck, Austria

**Keywords:** Cancer, Medical research, Oncology, Risk factors, Signs and symptoms

## Abstract

Febrile neutropenia secondary to chemotherapy is one of the most critical complications in cancer treatment. The aim of this study was to determine if an increase in the percentage of immature platelet fraction (IPF%) might predict early neutrophil recovery following cytostatic-dependent aplasia. A retrospective cohort study compared serial complete blood counts and the level of C-reactive protein (CRP) following induction chemotherapy for Ewing sarcoma and Non-Ewing sarcoma patients. The measurements were taken on a Sysmex XE-2100 instrument. A total of 287 paired samples from 28 children after the first cycle of chemotherapy were analyzed to test if an increase in the IPF% anticipated the CRP peak and recovery of neutrophil count. The chemotherapy associated nadir of neutrophils, reticulocytes and platelets was reached at 9.7 ± 1.5, 11.0 ± 1.7 and 11.9 ± 0.9 days (mean ± SD) respectively, in Ewing sarcoma patients. Still in severe neutropenia, IPF% was the first parameter that significantly increased and anticipated the CRP peak (11.9 ± 1.6 days, mean ± SD). The IPF% continuously increased (maximum = 6.56% ± 2.8%, mean ± SD) and peaked at 12.2 ± 1.4 days (mean ± SD) after commencement of chemotherapy. Compared to neutrophil recovery (14.6 ± 1.4 days, mean ± SD), the IPF% peak was anticipated by 2.4 days (p = 0.0085). Although variably treated, in non-Ewing sarcoma patients the effect was similar and the IPF% peak anticipated neutrophil recovery by 6.8 ± 4.7 days (p < 0.01). IPF% increased significantly at > 48 h before neutrophil recovery in patients treated with chemotherapy. IPF% is an inexpensive parameter and may be valuable in the management of febrile neutropenia.

## Introduction

Myelotoxicity—including neutropenia, thrombocytopenia and anemia—is the most frequent side-effect of chemotherapy in cancer patients^1^. Severe myelosuppression puts patients at risk of opportunistic and often fatal life-threatening infection, and is one of the major reasons for dose modifications, dose delays, or discontinuation of therapy^2^.

In the case of febrile neutropenia, early empirical antibiotic therapy is mandatory to avoid progression to sepsis. Antibiotics are continued until the patient is afebrile and clear signs of bone marrow recovery are detected^3,4^. When the neutrophil count remains low, controversy exists over whether antibiotic therapy should be stopped^5^. Discontinuation at uncertain blood culture results may put the patient at risk for sepsis progression with possibly fatal outcome. This is clearly of more concern than avoidance of drug-related adverse events or drug resistance^6^. Therefore, additional clinical parameters are urgently required for the management of antibiotic treatment.

Recent progress in understanding bone marrow physiology has identified immature platelets as a parameter of imminent bone marrow recovery after chemotherapy^7^. Immature platelets remain undetected in the traditional manual microscopic method. However, they can be measured by flow cytometry-based hematology analyzers^8^. The fact that the immature platelet fraction (IPF%) is high in patients with immune thrombocytopenia is already used to distinguish thrombocytopenia in acute lymphoblastic leukemia^9^. Although this value can be extrapolated to other scenarios, data on IPF% recovery after treatment with chemotherapy in children are sparse.

The aim of this study is to evaluate early IPF% recovery as an earlier indicator of absolute neutrophil recovery in the myelosuppressive phase of pediatric cancer patients in order to decide and control the length and intensity of a preemptive antibiotic therapy.

## Material and methods

### Patients and healthy controls

All 76 pediatric patients (age > 0 year to < 18 years) with newly diagnosed cancer and treated at our institution (Department of Pediatrics I, Medical University of Innsbruck, Austria) between January 2016 and July 2021 were recruited for the study. Fortyfive patients with the diagnosis of acute leukemia (39 acute lymphoblastic leukemia, 6 acute myeloid leukemia) were excluded from this study, because of severe bone marrow infiltration by tumor cells (27–99%) and chemotherapy was administered irrespective of white blood recovery (which is the standard practice in the induction phase). All other patients were monitored clinically for signs of infection at inpatient stay between the first and second cycle of chemotherapy. Complete blood sample and level of C-reactive protein (CRP) were drawn on a daily basis. The assignment to “febrile neutropenia” was fulfilled if a neutrophil count < 0.5 G/L and the presence of a single temperature > 38.3 °C or a sustained temperature of ≥ 38.0 degrees Celsius for more than one hour was present. Accordingly, three patients were excluded, because they did not fulill the criteria of “febrile neutropenia”. Finally, we collected data for 11 patients (Table 2) treated with the same therapy protocol (EWING 2008, consisting of neoadjuvant VIDE cycles and including vincristine 1.5 mg/m^2^ at day 1; ifosfamide 3.0 g/m^2^, doxorubicin 20 mg/m^2^ and etoposide 150 mg/m^2^, given on days 1, 2 and 3; the interval between two VIDE cycles was 21 days) and for 17 non-Ewing sarcoma patients with different diagnoses and treated with different protocols (Supplemental Table 1).

Normal IPF% reference values were measured in a collective of 416 healthy pediatric probands from November 2016 to March 2017 aged 6 months to 18 years (Table 1). Children were considered healthy if they were at the hospital for dietary, social, non-hematological, or elective surgery reasons.

**Table 1:** Characteristics in a healthy pediatric cohort of 416 patients according to IPF%, IPF# and MPV.

Parameter	0–1 y	1–10 y	10–18 y	p value
Number of healthy children	51	172	193	
Sex				
Male	29 (56.9%)	88 (51.2%)	92 (47.7%)	ns
Female	22 (43.1%)	84 (48.8%)	101 (52.3%)	
IPF%				
Mean [%] ± SD (median)	2.9 ± 1.8 (2.3)	1.7 ± 1.1 (1.5)	2.4 ± 1.4 (2.0)	0.0001*
Range [%] (min–max)	0.6–7.3	0.3–6.2	0.6–8.3	
IPF% percentiles				
90. P.	6.1	3.2	4.3	
95. P.	6.7	3.9	5.2	
IPF#				
Mean [G/L] ± SD (median)	11.3 ± 8.1 (9.5)	5.0 ± 2.9 (4.5)	5.7 ± 3.0 (5.1)	0.0001^#^
Range [G/L] (min–max)	3–55	1–19	1–17	
MPV				
Mean [fL] ± SD (median)	9.8 ± 0.8 (9.7)	9.5 ± 0.8 (9.4)	10.2 ± 0.8 (10.2)	ns
Range [fL] (Min–Max)	8.4–11.6	7.8–12.2	8.2–12.4	

IPF%, immature platelet fraction relative; SD, standard deviation; min, minimum; max, maximum; y, years; P., percentile, MPV, mean platelet volume; IPF#, immature platelet fraction absolut; ns, not significant.

*p value calculated across all age groups; 1–10 vs 10–18: p 0.0001, 1–10 vs 0–1: p 0.0001, 10–18 vs 0–1: p 0.072.

^#^p value calculated across all age groups; 1–10 vs 10–18: p 0.023, 1–10 vs 0–1: p 0.0001, 10–18 vs 0–1: p 0.0001.

### Laboratory methods

Routine venous blood samples from patients treated for Ewing sarcoma were collected in EDTA for blood cell analysis and in heparin for CRP level. Blood samples were planned daily from start of induction chemotherapy until bone marrow recovery and values were graded according to the CTCAE [Common Terminology Criteria for Adverse Events, Version 5.0, November 2017]. The following analyses were performed: leukocyte count, neutrophil count, level of hemoglobin, reticulocyte count, platelet count, percentage of immature platelets (IPF%), count of immature platelets (IPF#), and CRP level. Immediate and automatic measurement of immature platelets was performed using the Sysmex XE-2100 blood cell counter. Recovery of relative reticulocyte values was defined as levels > 2‰, recovery of neutrophils as levels > 0.5 G/L.

### Statistical analysis

Descriptive statistics were performed for all variables of interest, giving means, standard deviations, minimum and maximum ranges, and absolute and relative frequencies for qualitative variables. The chi-squared test was used for categorical variables and the Mann–Whitney U test for continuous variables. Data visualization and analysis was performed using GraphPad Prism, version 8.4, IBM SPSS Statistics, version 26, and Excel.

### Ethics

This study was performed in line with the principles of the Declaration of Helsinki. The Ethics Committee of the Medical University of Innsbruck approved the retrospective evaluation (EC No. AN2016-0059 360/4.10). All data were obtained from medical records. Waiver of informed consent given by Ethics Committee of the Medical University of Innsbruck.

## Results

### IPF% and IPF# count in age groups of healthy probands

IPF% reference values were collected on the basis of 416 healthy children and adolescents. The included probands were normally distributed between gender groups. The analysis included distribution of values and calculation of percentiles in various age groups as defined by the WHO classification of age groups (Table 1).

Significant differences between the three specified age groups were noted: 0–1 year infants, 1–10 years pre-school and primary school age children, 10–18 years adolescents. Infants showed higher mean and range values of IPF% (2.9% ± 1.8%, range 0.6–7.3%, versus 1.7% ± 1.1%, range 0.3–6.2%, p = 0.0001; Table 1) than did children of pre-school and primary school age (2.9% ± 1.8%, range 0.6–7.3%, versus 1.7% ± 1.1%, range 0.3–6.2%, p = 0.0001; Table 1). Furthermore, adolescents showed mean and range values of IPF% that differed from those of children of pre-school/primary school age (2.4% ± 1.4%, range 0.6–8.3%, versus 1.7% ± 1.1%, range 0.3–6.2%, p = 0.0001). Infants did not differ from the group of adolescents (10–18 years, p = 0.072). There was no gender difference for IPF% mean values (p = 0.24).

The IPF# differed in all three age groups in terms of mean and range values. Infants showed the highest values (11.3 G/L ± 8.1, mean ± SD and 3–55 range) as compared to pre-school/primary school age children (5.0 ± 2.9, mean ± SD and 1–19 range, p = 0.0001) and adolescents (5.7 ± 3.0, mean ± SD and 1–17 range , p = 0.0001). Compared to children of pre-school/primary school age, adolescents showed slightly higher IPF# values, which differed from the two other age groups (10–18 years versus 1–10 years: p = 0.023; 10–18 years versus 0–1 year: p = 0.0001, Table 1).

Regarding mean platelet volume we did not detect any difference between age groups (Table 1).

### Recovery of neutrophils, platelets and reticulocytes after chemotherapy

**Figure 1:**
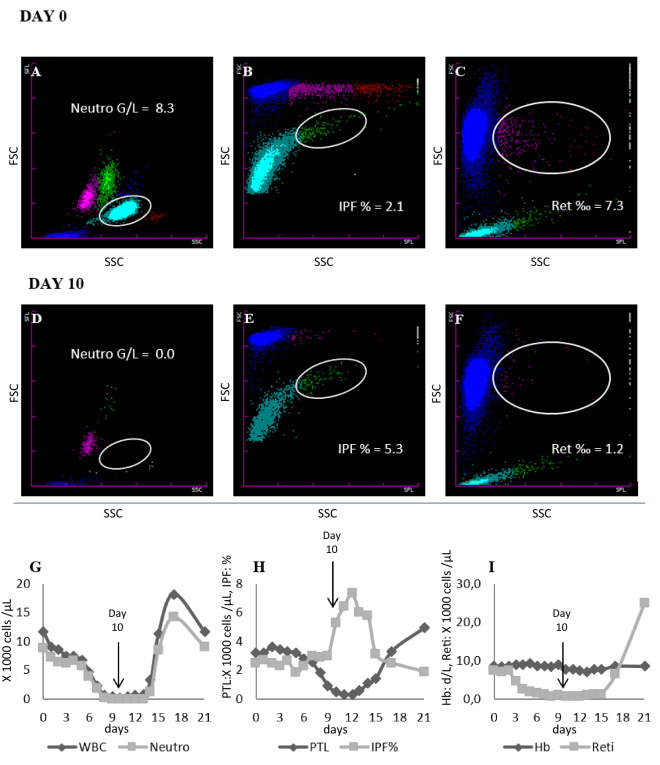
Exemplary patient from the Ewing sarcoma cohort: (**A**–**C**) neutrophil count, IPF%, and reticulocytes day 0; (**D**–**F**) neutrophil count, IPF%, and reticulocytes day 10 after chemotherapy application (nadir); (**G**–**I**) regeneration of neutrophil count, IPF%, and reticulocytes after day 10. A significant IPF% peak can be seen before the increase in neutrophils and erythrocytes.

Hematopoietic recovery during induction chemotherapy is illustrated based on one exemplary patient (16 years, female) during and after her first VIDE cycle (Fig. 1). On day 0, scattergrams (A–C) showed normal neutrophil values (8.3 G/L), IPF% (2.1%) and reticulocytes (7.3%o). Ten days after start of induction therapy (scattergrams D-F), neutrophil und reticulocyte counts were severely reduced (0.0 G/L and 1.2%o, respectively), but IPF% showed an increase to 5.3%. Finally, diagrams G-I show the further course of cell recovery: After a nadir of neutrophil count and reticulocytes on day 10, recovery of both cell lines occurred after 14 and 16 days, respectively (G and I). Diagram H illustrates the rise in IPF% on day 10 and the consecutive platelet recovery on day 13.

### Analysis of IPF% in Ewing sarcoma patients treated with chemotherapy

**Figure 2:**
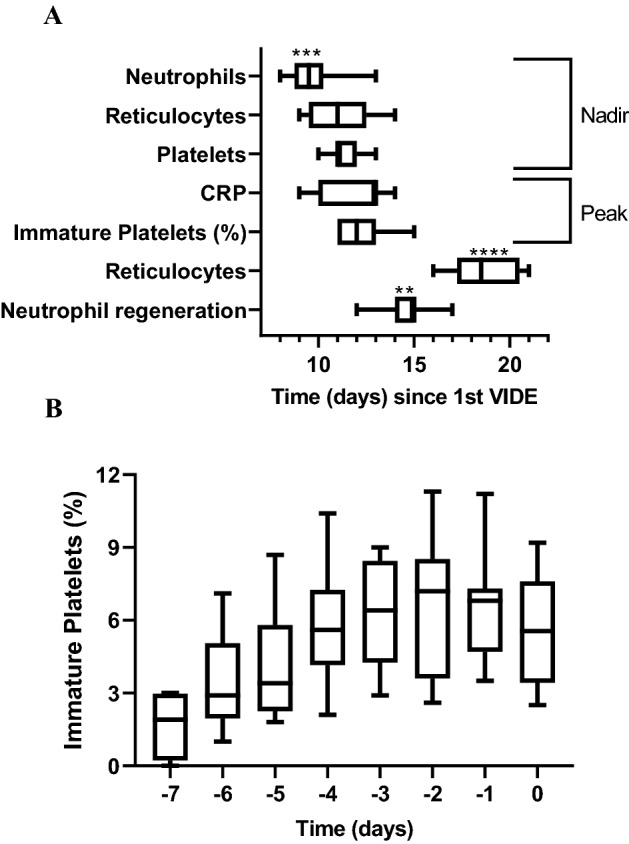
(**A**) Immature platelets (IPF%) have good potential for predicting reticulocyte (> 2‰, p < 0.0001) and neutrophil (> 0.1G/L, p = 0.0085) regeneration. The figure shows the nadir of neutrophils, reticulocytes, and platelets and the peak of CRP and IPF% after starting the first VIDE cycle (= day 0). The significant values relate to IPF%. (**B**) The IPF% peak occurs 2.4 days before regeneration of neutrophils (> 0.1G/L, day 0) and is 6.56% ± 2.8% (mean ± SD).

In order to investigate the predictive value of IPF%, we analyzed daily neutrophil, reticulocyte and thrombocyte count, the IPF%, the level of CRP and the presence of fever in samples from 11 patients treated for Ewing sarcoma. The patients were between 3.02 and 17.49 years old (median 14.01 years) and were equally distributed for sex and age (54.5% female, Table 2). Mean neutrophil, reticulocyte and platelet nadir was reached at 9.7 ± 1.5, 11.0 ± 1.7 and 11.6 ± 0.9 days (mean ± SD), respectively (Fig. 2a). At the same time, the patients developed fever (at 10.8 ± 1.8 days), CRP peak was noted (at 11.9 ± 1.6 days) and was followed by the IPF% peak at 12.2 ± 1.4 days (mean ± SD, Fig. 2a). Compared to the peripheral blood count recovery (> 0.5 G/L and > 2‰ for neutrophils and reticulocytes, respectively), the IPF% peak was predictive for reticulocyte (after 18.6 ± 1.8 days, mean ± SD; p < 0.0001) and neutrophil (after 14.6 ± 1.4 days, mean ± SD; p = 0.0085) regeneration (Fig. 2a). In fact, the IPF% peak (6.56% ± 2.8, mean ± SD) was observed two days before neutrophil regeneration (Fig. 2b).

**Table 2:** Patient cohort of 11 uniformely treated pediatric patients with newly diagnosed Ewing sarcoma.

Patient number	Age at diagnosis [y]	Sex	Localisation of primary tumor	BM involvement
#1	5.79	m	Os sacrum	No
#2	11.79	f	Lumbar vertebral arch	No
#3	11.61	f	Tibia diaphysis (sin., prox.)	Yes
#4	11.32	m	Femur diaphysis (dex.)	No
#5	17.49	f	Extraosseous Ewing	No
#6	16.42	f	Os ileum	No
#7	16.08	m	Extraosseous Ewing mamma (sin.)	No
#8	14.31	f	Os sacrum	No
#9	17.40	m	Fibula (sin., prox.)	No
#10	14.01	f	Femur diaphysis (dex.)	No
#11	3.02	m	Scapula (dex.)	No

y, years; m, male; f, female; sin., sinister; dex., dexter; prox., proximal.

### Analysis of IPF% in Non-Ewing sarcoma patients treated with chemotherapy

In order to extend the applicability of the findings to non-Ewing sarcoma, patients with different cancer diagnoses and variably treated were included (Supplemental Table 1). They also fulfilled the presence of chemotherapy associated febrile neutropenia. The non-Ewing sarcoma patients were between 0.01 and 16.08 years old (median 4.76, 1.0–5.75 IQ1-IQ3) and 10 (59%) of them were male. Mean neutrophil, reticulocyte and platelet nadir was reached at 10.2 ± 3.0, 6.2 ± 2.1 and 10.0 ± 3.5 days (mean ± SD). The IPF% peak was noted at 9.3 ± 2.8 days after start of chemotherapy. In 17 out of 17 patients the IPF% peak anticipated the neutrophil recovery, which was observed 6.8 ± 4.7 days later (p < 0.01).

## Discussion

In cancer patients, myelotoxicity is the most frequent side-effect of cytostatic therapy^1^; it puts the patient at risk for developing septic or bleeding complications and often results in delays in planned chemotherapy^2^. For this reason, understanding the mechanism of bone marrow toxicity is an important step toward improving supportive care in cancer treatment. Measurement of platelet RNA offers the opportunity to quantify the process of bone marrow regeneration after chemotherapy^10–13^. In fact, automatic hematology analyzers detect immature platelets easily and their quantification correlates with the rate of thrombopoiesis^10–12,14–17^. At first glance, it is not obvious how thrombopoiesis plays a role in predicting bone marrow recovery, but IPF% has already been recognized as a parameter of imminent thrombocyte regeneration following cytostatic treatment^18^. Whether the data on IPF% can predict the rise in other peripheral blood cells after chemotherapy is still unknown.

The example given in Fig. 1A–I shows the typical course of peripheral blood cell counts in a patient after initial cytoreductive treatment. The optimal management in this situation requires a detailed anamnesis and physical examination—with particular attention given to the skin, catheter sites, lungs, sinuses, mouth and abdomen. In the case of a patient with unclear fever and neutropenia, immediate empiric therapy with a dose of intravenous antibiotics is required. Once the patient is improving and blood cultures remain negative, treatment can be de-escalated to more narrow coverage. Whether the patient is continued on this therapy before neutropenia has resolved, is controversial and practices vary among centers if no infectious etiology is identified. Conversely, the responsible clinician will closely evaluate the patient for signs of neutrophil recovery. In this example we demonstrate that an increase in IPF% anticipates regeneration of neutrophilic granulocytes and suggests that discontinuation of antibiotics before neutrophil recovery in a stable patient with recognition of increasing IPF% may be a reasonable approach.

Although we anticipate that more data on this topic are necessary, a much broader investigation of the normal levels of IPF% is needed. Numerous studies limit the analysis of IPF% to healthy and adult patients and show values between 0.3 and 17.8%^19^. The most accurate studies are performed in accordance with the CLSI (Clinical and Laboratory Standards Institute) guidelines in very large study populations and result in IPF% values between 0.5–3.2% and 0.4–3%^20^. In pediatrics, large studies in different age groups are missing. One single study limits the analysis to the neonatal period and attributes distinct values to different gestational ages, with the values differing between 1.5 and 5.9%^19^. Another study limits the IPF% values (0.7–5.7%) to 100 children between 6 months and 18 years of age and includes samples from patients with a wide variety of different diagnoses^9^. Our analysis of IPF% in 416 healthy children shows that IPF% is sex-independent and differs concerning the age (Table 1). Infants have the highest IPF% in comparison to other age groups. Although the comparison is made with a small number of patients, this correlates with previous data showing higher IPF% in children younger than 4 years of age^9^. It is known that thrombopoietin has higher concentration levels in this age group, which translates to increased activity of megakaryocytes and production of thrombocytes^9,21,22^.

Considering that IPF% is a useful diagnostic parameter for identifying the production of thrombocytes^8,12^, it has long been claimed to have its value in predicting neutrophil regeneration after chemotherapy^23^. After observing the course of cell count in an individual case, we analyzed 11 uniformly treated patients diagnosed with Ewing sarcoma. The nadir of neutrophil, reticulocyte and platelet count resulted early after initiation of chemotherapy and was immediately paralleled by a simultaneous rise in IPF%. Interestingly, in our study the IPF% peak occurred almost simultaneously with the CRP peak (Fig. 2a). The increase of CRP has a high latency and the decrease of CRP levels is delayed because of its long half-life (25–30 h), resulting in a poor utility as a predictive value. In contrast, increase of IPF% anticipates remission from infection, because it indicates bone marrow recovery. Identical results in non-Ewing sarcoma patients with a broad variety of underlying disease and who received different cytostatic drugs expand the applicability of the observed findings. In all cases, the increase in peripheral blood cells followed a little later, in the order of platelets, neutrophilic granulocytes and reticulocytes. This sequence makes sense for the expected regeneration of bone marrow after chemotherapy, as platelets have the shortest proliferation time and reticulocytes have the longest time. On the other hand, caution is advised for this interpretation, as platelets are destroyed and IPF% is subsequently increased in patients with sepsis, regardless of the treatment with chemotherapy^16,24,25^. In this sense IPF% has been proposed as a biomarker for the prediction of sepsis diagnosis and severity^26–28^.

Nonetheless, the findings presented and discussed here must be interpreted with caution and a number of limitations should be borne in mind. Most of the studies, including ours, were performed retrospectively and possibly include a biased selection of controls. Our and other studies may underestimate the IPF% values, as this parameter appears very quickly and disappears at the same time. Furthermore, IPF% measurement is not standardized and is performed with different devices. The only way to overcome these limitations is to perform a prospective clinical study with a large number of subjects, well-defined methods and precisely formulated aims.

We conclude that analysis of IPF% may give helpful data on neutrophil activity and kinetics in the post-chemotherapy period in children. Considering these data may help determine timing and the need for antibiotic therapy in phases of aplasia. We suggest that this method be included in routine report of blood differential in pediatric oncology, as the barriers to integrate IPF% are minimal—it is not expensive, readily available and it is a precise method for estimating bone marrow kinetics. Finally, prediction of bone marrow recovery can facilitate overall survival, as a more rapid application of chemotherapy is associated with improved survival^29–32^.

## Supplementary Information

Supplementary Table 1.

## Data Availability

The datasets used and/or analysed during the current study are available from the corresponding author on reasonable request.
