# Supplementary material for: Immature platelet fraction predicts early marrow recovery after severe chemotherapy associated neutropenia
Source: Sci Rep. 2023 Feb 27;13:3371. doi: 10.1038/s41598-023-30469-3 (PMC9971198; doi:10.1038/s41598-023-30469-3)
Supplement: Supplementary file 1 — Supplementary Table 1. [file 41598_2023_30469_MOESM1_ESM.pptx]

## Slide 1
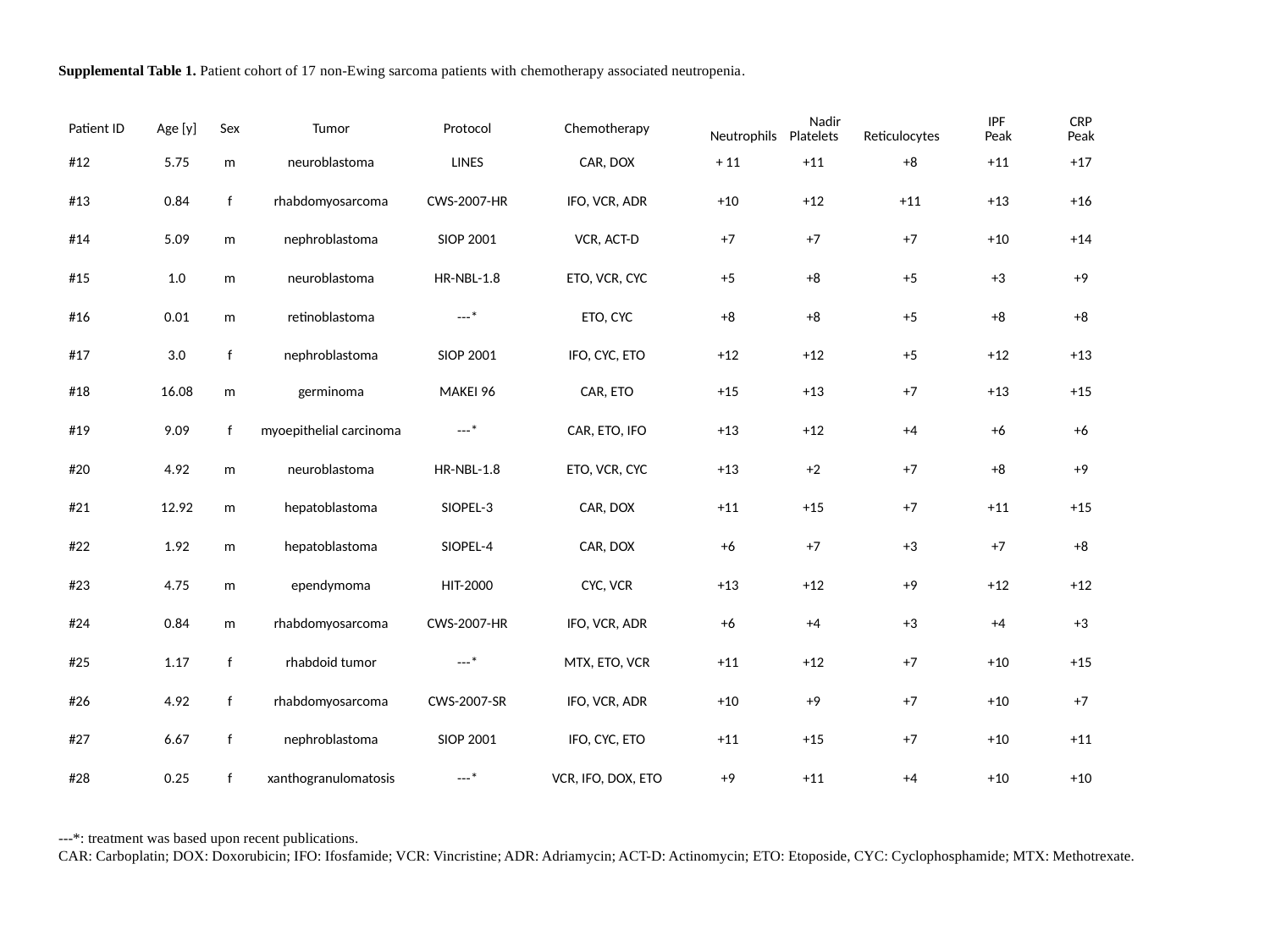

Supplemental Table 1. Patient cohort of 17 non-Ewing sarcoma patients with chemotherapy associated neutropenia.
| Patient ID | Age [y] | Sex | Tumor | Protocol | Chemotherapy | Nadir Neutrophils Platelets Reticulocytes | | | IPF Peak | CRP Peak |
| --- | --- | --- | --- | --- | --- | --- | --- | --- | --- | --- |
| #12 | 5.75 | m | neuroblastoma | LINES | CAR, DOX | + 11 | +11 | +8 | +11 | +17 |
| #13 | 0.84 | f | rhabdomyosarcoma | CWS-2007-HR | IFO, VCR, ADR | +10 | +12 | +11 | +13 | +16 |
| #14 | 5.09 | m | nephroblastoma | SIOP 2001 | VCR, ACT-D | +7 | +7 | +7 | +10 | +14 |
| #15 | 1.0 | m | neuroblastoma | HR-NBL-1.8 | ETO, VCR, CYC | +5 | +8 | +5 | +3 | +9 |
| #16 | 0.01 | m | retinoblastoma | ---\* | ETO, CYC | +8 | +8 | +5 | +8 | +8 |
| #17 | 3.0 | f | nephroblastoma | SIOP 2001 | IFO, CYC, ETO | +12 | +12 | +5 | +12 | +13 |
| #18 | 16.08 | m | germinoma | MAKEI 96 | CAR, ETO | +15 | +13 | +7 | +13 | +15 |
| #19 | 9.09 | f | myoepithelial carcinoma | ---\* | CAR, ETO, IFO | +13 | +12 | +4 | +6 | +6 |
| #20 | 4.92 | m | neuroblastoma | HR-NBL-1.8 | ETO, VCR, CYC | +13 | +2 | +7 | +8 | +9 |
| #21 | 12.92 | m | hepatoblastoma | SIOPEL-3 | CAR, DOX | +11 | +15 | +7 | +11 | +15 |
| #22 | 1.92 | m | hepatoblastoma | SIOPEL-4 | CAR, DOX | +6 | +7 | +3 | +7 | +8 |
| #23 | 4.75 | m | ependymoma | HIT-2000 | CYC, VCR | +13 | +12 | +9 | +12 | +12 |
| #24 | 0.84 | m | rhabdomyosarcoma | CWS-2007-HR | IFO, VCR, ADR | +6 | +4 | +3 | +4 | +3 |
| #25 | 1.17 | f | rhabdoid tumor | ---\* | MTX, ETO, VCR | +11 | +12 | +7 | +10 | +15 |
| #26 | 4.92 | f | rhabdomyosarcoma | CWS-2007-SR | IFO, VCR, ADR | +10 | +9 | +7 | +10 | +7 |
| #27 | 6.67 | f | nephroblastoma | SIOP 2001 | IFO, CYC, ETO | +11 | +15 | +7 | +10 | +11 |
| #28 | 0.25 | f | xanthogranulomatosis | ---\* | VCR, IFO, DOX, ETO | +9 | +11 | +4 | +10 | +10 |
---*: treatment was based upon recent publications.
CAR: Carboplatin; DOX: Doxorubicin; IFO: Ifosfamide; VCR: Vincristine; ADR: Adriamycin; ACT-D: Actinomycin; ETO: Etoposide, CYC: Cyclophosphamide; MTX: Methotrexate.
